# Editorial: Somatosensory Integration in Human Movement: Perspectives for Neuromechanics, Modelling and Rehabilitation

**DOI:** 10.3389/fbioe.2021.725603

**Published:** 2021-07-14

**Authors:** Leonardo Gizzi, Ivan Vujaklija, Massimo Sartori, Oliver Röhrle, Giacomo Severini

**Affiliations:** ^1^Institute for Modelling and Simulation of Biomechanical Systems, Chair for Continuum Biomechanics and Mechanobiology, University of Stuttgart, Stuttgart, Germany; ^2^SimTech Cluster of Excellence “Data Integrated Simulation Science” EXT 2075, University of Stuttgart, Stuttgart, Germany; ^3^Department of Electrical Engineering and Automation, Aalto University, Espoo, Finland; ^4^Department of Biomechanical Engineering, University of Twente, AE Enschede, Netherlands; ^5^School of Electrical and Electronic Engineering, University College Dublin, Dublin, Ireland

**Keywords:** somatosensory integration, motor control, neurorehabilitation, human machine interface, visuomotor integration, locomotion, myoelectric control, vibrotactile feedback

Paraphrasing Sherrington (1924), humans interact and engage with their environment solely through movement. They move thanks to a series of coordinated muscles contractions ultimately driven by the firing of alpha motor neurons in the spinal cord. The activity of these neurons, emerges as the result of the interplay between the motor commands generated across the central nervous system (CNS) and the information about the state of the body and of the environment (Enoka, 2015). The latter, harvested by a multitude of different sensory organs. The integration of efferent and afferent information determines, at the level of the motor neuron, whether or not an action potential will be produced at any point in time (Kandel et al., 2000). Sensory information influences also the behavior of several other structures across the central nervous system. An alteration of the sensory organs (or neurons) themselves, or of the interpretation that the CNS makes of the information they provide, can have profound consequences on the aptness of an individual to carry out a motor task. This has obvious repercussions on their capability of interacting with the environment. Taken together, those considerations suggest that research on somatosensory integration should follow an interdisciplinary approach, whose methods range from experiments to modeling and whose topics span from basic physiology to rehabilitation, from prosthesis control to biologically-inspired robotics, from human-computer interaction to ergonomics.

When we first opened the Topic for submissions, in 2019, we could not imagine that 115 authors would enthusiastically respond to our call, contributing with 21 original manuscripts that tackled the sensorimotor integration problem from different, yet complementary, perspectives. The contributors proposed new experimental approaches for a selective and controlled alteration of sensory information, development of sensation-aware neuromusculoskeletal models, and the evaluation of sensory integration, substitution, or function replacement in the context of human-machine communication.

The manuscripts have been divided in four categories, the most populated concerning Embodiment/Proprioception and Locomotion, to provide an immediate overview of the Research Topic.

## 1. Embodiment, Perception, Feedback

### 1.1. Visual Information Integration

Vision is the primary sensory modality for movement, as reflected by the fact that the motor plan can be effectively and systematically perturbed by altering the relationship between the movement environment and the associated visual feedback. A clear example of this phenomenon, is the visuomotor rotation paradigm (Krakauer, 2009), where a visual rotation of the feedback of a movement generates a congruent rotation of the way in which the movement is generated. Here Severini and Zych showed that the rotation between visual feedback and motor output results in a congruent, but greedy, rotation of the activation of the muscle synergies associated with the task. They showed that the synergies activations are selectively rotated if the visual perturbation engages the workspace of a synergy at its boundaries and a rotation is needed for successfully reaching the target. This phenomenon could be behind the adaptation generalization behaviors previously observed at the muscle synergies level (De Marchis et al., 2018). Castronovo et al. have further shown the primacy of visual feedback over proprioceptive feedback in the same visuomotor rotation paradigm.

It has been long known that small currents applied to the muscles can improve motor performance, especially during challenging standing tasks (Severini and Delahunt, 2018). This is supposedly caused by the stochastic resonance phenomenon, where a small noise can improve detection of weak, undetectable, signals (in this case, small signals from proprioceptive afferents). Here, the authors show that this improvement in motor performance through sub-sensory stimulation is observable also during isometric reaching exercises—when visual feedback is not present—but disappears when visual feedback is available, suggesting a primacy of this latter sensory modality over proprioceptive information. This phenomenon is reflected also in the occurrence of cortical damages. As shown by Iosa et al. stroke patients heavily rely on visual information for the execution of hand movements. Visual feedback however, may not be sufficient to alter the internal model that the central nervous system builds of the environment. In fact, Lascaleia et al. simulated, through virtual reality, the fall or roll of a spherical object in different gravity conditions. They reported that only in earth-like gravity conditions the participants were able to correctly anticipate the position of the object at a specific point in time. They authors concluded that the CNS relies on an internal model of Earth gravity effects.

### 1.2. Proprioception and Vibrotactile Feedback

Proprioception is the sense of body position and motion, and is often referred to as “kinaesthesia.” It is useful to navigate environments and interact with objects, and represents a discrete and intuitive channel to foster neural plasticity and bi-directionally communicate with patients in the general context of human-machine interaction.

Movement emerges from the integration of efferent commands and afferent feedback. As shown here by Hu et al., it is possible to use the recorded neuromechanical output (i.e., high-density EMG and dynamometrics) to predict limb mechanics in unaffected individuals. However, if the quality of afferent feedback is altered—like in the blood flow restriction experimental protocol proposed by Gizzi et al.—the neural drive to the muscle is compensated to grant the desired mechanical output, and therefore needs to be estimated accordingly. It is worth noting, however, that an alteration of the neural pathways responsible for proprioception, can also take place far from the sensory areas themselves. With that regard, Pilkar et al. have shown that even when the lesion is central (cortical) and not peripheral, a diminished sensory acuity can be recorded, that has direct influence on the capability of the subjects to recover from a mechanical perturbation.

With the aim of eliciting neural plasticity, proprioceptive feedback was stimulated via skin vibration in the work of Asín-Prieto et al.. The authors, proposed a gamified, haptic, adaptive feedback loop, to promote motor learning during training with a robotic ankle exoskeleton. They reported an increased cortico-spinal excitability and improved game scores, in healthy individuals and one stroke patient after three sessions.

Ballardini et al. show here that vibrotactile feedback can be used as an additional sensory modality to the ones already available during a standing task, and that this additional feedback can improve task performance if made to encode task-relevant information.

Similarly, Ding et al. reported that mirror visual feedback combined with vibrotactile stimulation can facilitate the embodiment perception, compared to visual feedback alone. Embodiment perception is crucial for a successful recovery in stroke patients. The authors attributed this effect to an increased motor cortical activation.

A better understanding of somatosensory feedback is necessary to increase the efficiency of human-machine communication. In their work, Nataletti et al. explore the limits of electrotactile stimulation, concluding that temporal patterns are more relevant than the total energy or the duration in judging the numerosity of stimulation points.

## 2. Locomotion

Somatosensory integration plays a central role during locomotion. Mileti et al. have shown, similarly to Oliveira et al. (2016), that subtle changes in sensory perception during locomotion, such as those happening when walking on a treadmill vs. when walking overground, can lead to more stereotypical recruitment of the muscle synergies involved in the task. This result suggests that small changes in sensory feedback during locomotion, although not affecting the muscular co-activation strategies, can lead to alterations in the neural strategies of task execution. Integrating then different feedback modalities during gait training of neurologically impaired individuals may prove crucial in improving therapy results. As a point in case, here Tan et al. show that robot-based gait training providing guidance and haptic feedback during walking to sub-acute stroke survivors, can lead to changes in synergies activations (as opposed to what found earlier on chronic patients, Gizzi et al., 2011) that reflect in more symmetrical activation patterns with respect to standard therapy.

Furthermore, exposing patients to carefully tailored perturbations and the consecutive observation of the motor adaptations emerging due to the inherent somatosensory integration, can be used to aid the rehabilitation process (Reisman et al., 2013). Essentially, altering specific gait parameters in patients with motor impairments, following a given rehabilitation intervention, can lead to more informed conclusions on individual long-term stability of locomotion (Cajigas et al., 2017). To further enhance this process, biofeedback can be used to deliver desired gait perturbations in a more targeted manner. In fact, Torricelli et al. have shown here that guided voluntary perturbation through EMG based visual biofeedback- results suggest that can lead to short-term learning in rhythmic tasks. This learning is built upon synergistic temporal commands that are robust to changes in the task demands.

The latter, develop from hard-wired networks that are shared across species with a common evolutionary ancestor (see, for example Dominici et al., 2011), and reach maturity (in humans), after a few years. Cappellini et al. reviewed the maturation of the locomotor circuitry in cerebral palsy (CP) children, suggesting that early training for central pattern generator-modulating therapies are necessary and indicating robotic aid-based therapy challenge of the discussed human-machine interfaces as an important player.

## 3. Human-Machine Interfacing

Enhancing efficacy of rehabilitation technologies through use of engaging human-machine interfaces, has been an ongoing research pursuit (Lagoda et al., 2012; Vujaklija, 2018). To that end, Electromyography (EMG) and Electroencephalography (EEG) have been the two most considered interfacing modalities, as they offer a chance for an intimate observation of the underlying neural activity while remaining non-invasive and minimally intrusive (Farina et al., 2021). However, establishing robust controllers of assistive devices based on these interfaces, is difficult and many of the proposed approaches have failed to meet their clinical promises. One reason for this, might lie in the discrepancy between the laboratory tests and the real-world conditions. In fact, it has been shown that commonly considered laboratory indicators of performance of the advanced prosthetic EMG interfaces are poor predictors of clinical outcomes (Vujaklija et al., 2017). This is likely since these offline metrics consider performance separately from the active user and thus do not account for any active and/or dynamic changes that occur during the online human-machine interaction.

To overcome these drawbacks, in addition to real-time experiments, inclusion of more descriptive data collection has been considered. For instance, real-time control of prosthetic hands has benefited from training data that accounts for biosignals gathered during multiple steady-state arm positions (Amsuess et al., 2015). Moreover, here Gigli et al. have taken this approach a step further, in order to make the procedure faster and less tiresome. Namely, they have collected the algorithm training data from users as they were moving their arm smoothly through multiple postures and have shown that the same improved results can be obtained in shorter time.

Another outstanding challenge of the discussed human-machine interfaces is the lack of bi-directionality. Namely, in the past two decades there has been a growing interest in using BCI technology for closing the loop between movement intent and the feedback provided by assistive devices. In this context, BCI technology can be used to trigger the assistive device based on the motor imagery of the target movement, and the device provides somatosensory feedback on the movement, thus reinforcing the afferent/efferent connection. Guggenberger, Raco et al. analyzed in depth the differences in workload (e.g., mental, temporal, effort-related) required by individuals while using two different assistive devices (robot vs. functional electrical stimulation) driven by a BCI system during finger extension movements. Guggenberger, Heringhaus et al. also investigated how feedback based on neuromuscular stimulation modulates the gain of the spinal motor output. This results highlight the necessity of considering the effect of both afferent and efferent information when designing BCI-based feedback systems.

Similarly, Ortiz et al. proposed a novel brain-machine interface based on gamma-band EEG and attention level that showed encouraging results for controlling a lower-limb exoskeleton in intact individuals.

## 4. Modeling

Computational models of the composite neuro-musculo-skeletal system, allow investigating the neuro-mechanical interplay underlying movement in the controlled and predictive environment of simulation (Klotz et al., 2020). This provides new tools to determine the relative weight of the different components in the neuromusculoskeletal system, that determine the final motor output (Sartori et al., 2017). As previously reported, sensory information is especially difficult to harvest in intact moving humans, and physiologically correct, person-specific neuro-mechanical simulations represent a viable contribution to advance toward more complete hypotheses on how the central nervous system control physical movement in a closed-loop fashion (Sartori and Sawicki, 2021).

With their *in-silico* experiments, Stollenmeier et al. showed that it is possible to use neuromusculoskeletal models to accurately predict the effects of static and dynamic perturbations on the motion of the human upper limb. The proposed approach integrated both short and long latency reflexes, to represent the spinal and supraspinal (reflex) contributions to movement. This underlay the importance of somatosensory integration in the resulting motor output, in an active environment.

Similarly, Koelewijn and Ijspeert have proposed a neuromusculoskeletal model to represent the sagittal reactions to ground perturbations during standing. Also in this case, the model comprised spinal reflexes. They reported that the model results were poorly to moderately correlated with experimental results, if the control optimization aimed at minimizing the subject's effort. This suggests that other factors (we hypothesize here proactive stabilization of the joints by mean of increased muscle stiffness), may play a more central role than energy efficiency.

## 5. Conclusion

Somatosensory (and, more in general, afferent) action potentials are elusive and difficult to harvest in humans, but may represent a key factor in understanding human neuromechanics as well as in creating more effective human-machine interaction and rehabilitation technologies. A paradigm shift in experimental procedures is necessary to generate more comprehensive theories of afferent integration in the motor system. The experimental and modeling efforts testified by this collection, show that the topic is very much alive and provide a solid and promising basis for future developments.

## Author Contributions

LG, IV, and GS drafted the first version of the manuscript. All authors contributed in the critical discussion and revision of its contents.

## Conflict of Interest

The authors declare that the research was conducted in the absence of any commercial or financial relationships that could be construed as a potential conflict of interest.
